# 

**Published:** 1889-04

**Authors:** 


VOL. X.] PUBLISHED QUARTERLY AT TWO DOLLARS A YEAR. SINGLE COPIES, SIXTY CENTS. [NO. 2.

### THE JOURNAL OF COMPARATIVE MEDICINE AND SURGERY.

LIBRARY INDEXED S.G.O.

EDITED BY

W. A. CONKLIN, Ph. D., D. V. S.,

DIRECTOR OF ZOOLOGICAL GARDENS, NEW YORK CITY.

RUSH SHIPPEN HUIDEKOPER, M. D., Veterinarian (*Alfort*),

DEAN OF VETERINARY DEPARTMENT, UNIVERSITY OF PENNSYLVANIA.

COLLABORATORS.

COMPARATIVE ANATOMY AND PHYSIOLOGY.

PROF. JOSEPH LEIDY, M.D., L.L.D., . University of Penna.
PROF. HENRY C. CHAPMAN, M.D., . Jefferson Med. College.
PROF. E. C. SPITZKA, M.D., . . . . . . . New York City.
PROF. R. MEADE SMITH, M.D., . . . University of Penna.
PROF. J. T. DUNCAN, M.D., V.S., . Ontario Veterinary College.
PROF. C. M. O'LEARY, M.D., L.L.D., . . . New York City.

COMPARATIVE PATHOLOGY.

HENRY O. MARCY, M.D. . . . . . . . Boston, Mass.
PROF. JAMES TYSON, M.D., . . . . University of Penna.
PROF. R. H. FITZ, M.D., . . . . . . Harvard University.
PROF. WM. OSLER, M.D., . . . . . University of Penna.
E. O. SHAKESPEARE, M.D., . . . . Blockley Hospital, Phila.
A. W. CLEMENT, V. S., . . . . . . . Montreal Vet. College.
PROF. A. H. BAKER, V. S., . . . . . . . Chicago Vet. College.

COMPARATIVE SURGERY.

PROF. JAMES LAW, F.R.C.V.S., . . . . Cornell University.
PROF. C. P. LYMAN, F.R.C.V.S., . . . Harvard University.
PROF. D. McEACHRAN, F.R.C.V.S., . . Montreal Vet. College.
PROF. A. T. YOKURA, V.S., . . Imperial School, Tokio, Japan.
PROF. JAMES L. ROBERTSON, M.D., V.S., Am. Vet. College.
PROF. WILLIAM L. ZUILL, M.D., D.V.S., University of Penna.

A. L. HUMMEL, M.D., PUBLISHER,

No. 224 South Sixteenth Street, Philadelphia, Pa.

LONDON: BALLIERE, TINDALL & COX.

